# A novel double antibody sandwich-lateral flow immunoassay for the rapid and simple detection of hepatitis C virus

**DOI:** 10.3892/ijmm.2012.1121

**Published:** 2012-09-06

**Authors:** TINGXIU XIANG, ZHENG JIANG, JIAN ZHENG, CHAOYU LO, HARRY TSOU, GUOSHENG REN, JUN ZHANG, AILONG HUANG, GUOQI LAI

**Affiliations:** 1 Molecular Oncology and Epigenetic Laboratory and; 2 Department of Gastroenterology, The First Affiliated Hospital of Chongqing Medical University;; 3 Key Laboratory of Molecular Biology on Infectious Diseases, Ministry of Education, Chongqing Medical University, Chongqing, P.R. China;; 4 Artron BioResearch Inc., Burnaby, BC, Canada

**Keywords:** hepatitis C virus, recombinant protein, double antibody sandwich-lateral flow immunoassay, colloidal gold

## Abstract

The objective of this study was to screen for antigens of the hepatitis C virus (HCV) to establish a new double antibody sandwich-lateral flow immunoassay (DAS-LFIA) method for testing the presence of anti-HCV antibodies in human serum or plasma. A series of different recombinant HCV proteins in *Escherichia coli* cells were constructed, expressed, purified and the new DAS-LFIA strip was developed. The sensitivity and specificity of new the DAS-LFIA strip were evaluated by detecting 23 HCV-positive sera, a set of quality control references for anti-HCV detection that contain known amounts of anti-HCV antibodies, and 8 HCV-negative sera. A total of 300 clinical serum samples was examined by both the new DAS-LFIA strip and enzyme-linked immunosorbent assay (ELISA). Data were analyzed using SPSS 11.5 software. The sensitivity and specificity of the new DAS-LFIA strip were 100%. The lowest test line of the HCV DAS-LFIA strips was 2 NCU/ml. Additionally, the concordance between the new DAS-LFIA strip and ELISA methods was 94.33%. In conclusion, our new testing method is rapid, simple, sensitive and specifically detects the presence of anti-HCV antibodies in human serum or plasma. Therefore, it may be used for monitoring HCV.

## Introduction

It is estimated that approximately 180 million people have been infected with the hepatitis C virus (HCV) and approximately 130 million people are chronic HCV carriers (1). HCV is a common cause of chronic hepatitis, cirrhosis and hepatocellular carcinoma worldwide (2–4). Currently, there are two main methods for detecting an HCV infection: one detects viral RNA by RT-PCR (5–8) and the other detects HCV antibodies by immunoassay [enzyme-linked immunosorbent assay (ELISA)] in serum (9,10). The sensitive ELISA assay uses recombinant viral proteins corresponding to multiple polypeptides from different viral regions, including structural proteins and non-structural polypeptides (11–13). It can be used as a confirmation test. However, its usage is limited in the clinical setting as the procedure requires sophisticated laboratory equipment and there is a high probability of contamination. Therefore, the purpose of our study was to develop an affordable and reliable rapid lateral flow test to detect the presence of HCV antibodies in blood samples by screening for HCV antigens, which would help decrease the chances of HCV infection from blood transfusions.

## Materials and methods

### Plasmids and bacterial strains

The p90/HCVFLlongpU plasmid carrying full-length coding sequences of HCV was a generous gift from Professor Charles M. Rice from the Center for the Study of Hepatitis C, Rockefeller University, New York, NY, USA. The *Escherichia coli* (*E. coli)* strains, Jm109, DH5a and BL21 (DE3), were used as the cloning and expression hosts.

### Reagents and instruments

A panel of 23 standard positive sera, 8 standard negative sera, a set of quality control references for anti-HCV detection that contain known amounts of anti-HCV antibodies (Artron BioResearch Inc., Burnaby, BC, Canada) and 300 clinical sera were used for the antigenicity assessment of HCV proteins. Other reagents and instruments included goat anti-mouse HCV IgG polyclonal antibody, 30–60 nm colloidal gold particles (from Artron BioResearch Inc.), HCV-ELISA (KHB, Shanghai, China), a NanoDrop^®^ ND-1000 Spectrophotometer, a Bio-Rad BioLogic LP, ZQ4000 test strip cutter, and XYZ-3000 Bio-Dot (all from Bio-Rad, Shanghai, China).

### Construction and expression of recombinant HCV antigens

**Figure 1.**
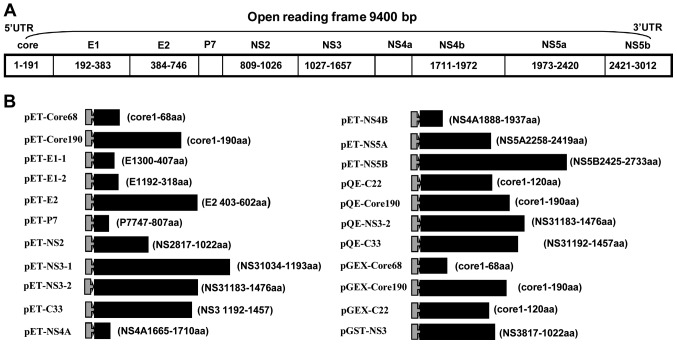
HCV genome and recombinant proteins. (A) The HCV genome contains a single major open reading frame (ORF) flanked by untranslated regions (UTRs). The 10 proteins encoded within the main ORF are indicated by alternated shading. (B) Genomic fragments of HCV were cloned into the prokaryotic expression vectors, pET32a(+), pQE30, or pGEX-4T-2, in-frame downstream of the 6-His-tag or glutathione S-transferase (GST)-tag coding sequence.

To obtain the HCV antigens, the sequences encoding the desired regions in the HCV genome were amplified by RT-PCR, and cloned into the prokaryotic expression vectors, pQE30 (Qiagen, Hilden, Germany), pET32a(+) (Novagen, Darmstadt, Germany), or pGEX-4T-2 (GE Healthcare Life Sciences, Chalfont St. Giles, UK), in-frame downstream of the 6-His-tag or glutathione S-transferase (GST)-tag coding sequence. Primers used for the HCV PCR amplification are listed in Table I and the structures of the plasmids are illustrated in Fig. 1. *E. coli* BL21 (DE3) cells harbouring the HCV gene fragment were grown at 37°C in LB medium containing 50 μg/ml of ampicillin to OD_600_ = 0.8. The expression of the antigens was induced by adding isopropyl-β-D-thiogalactopyranoside (IPTG) to a final concentration of 1 mmol/l. The cells were harvested 4–6 h later by centrifugation at 10,000 rpm for 15 min and stored at −20°C. Solubility analyses of expression products were performed as previously described (14). Briefly, harvested bacteria were re-suspended in phosphate-buffered saline (PBS; containing 140 mmol/l NaCl, 2.7 mmol/l KCl, 10 mmol/l Na_2_HPO_4_, 1.8 mmol/l KH_2_PO_4_, pH 7.3), sonicated on an ice-bath, and centrifuged at 10,000 rpm for 20 min at 4°C. After centrifugation, the soluble and insoluble fractions were analyzed for the presence of expression products.

### Purification of recombinant HCV antigens

To purify the expressed proteins, we chose to use a Ni-nitrilotriacetic acid (Ni-NTA) affinity chromatography column for His-tagged proteins and a glutathione sepharose™ 4B column for GST-tagged proteins. The two methods are similar as regards experimental procedures but differ in column chromatography and reagents, as described below:

*i) Protein purification with Ni-NTA column.* The column was first equilibrated with lysis buffer (50 mM NaH_2_PO_4_, 300 mM NaCl, 10 mM imidazole, pH 7.8) at five times the volume of the beads. The sample was then loaded and allowed to flow slowly in order to maximize the amount of protein bound to the beads. The flow-through solution was collected for SDS-PAGE analysis later. After the sample was completely loaded, washing buffer (50 mM NaH_2_PO_4_, 300 mM NaCl, 20 mM imidazole, pH 8.0) was added to wash off unspecific proteins bound to the beads or remaining in the column until the OD_280_ reading was below 0.100. The proteins of interest were eluted by adding elution buffer (50 mM NaH_2_PO_4_, 300 mM NaCl, 250 mM imidazole, pH 8.0).

*ii) Protein purification with glutathione sepharose 4B column.* The general procedures were the same as those for the Ni-NTA columns, although the buffers used in the GST columns were different. PBS solution (pH 7.4) was used for equilibration. After the sample was loaded, the same PBS solution was used to wash the column. Finally, the glutathione solution (50 mM Tris-HCL, 10 mM reduced glutathione) was used to elute the protein of interest. All purified recombinant HCV antigens were found to be >90% pure based on SDS/polyacrylamide gel analysis followed by Coomassie blue staining. Eluents with high OD_280_ were collected into a membrane bag and dialysis was performed overnight at 4°C in a buffer (PBS buffer). Protein concentration was measured using the Bradford method with bovine serum albumin as the standard.

### Construction of double antigen sandwich-lateral flow immunoassay (DAS-LFIA) strip and indirect lateral flow immunoassay (I-LFIA)

The DAS-LFIA device for the detection of anti-HCV antibodies was manufactured by Artron BioResearch Inc. First, we optimized the conditions for the colloidal gold conjugation of the purified antigen and the coating of the gold-conjugated recombinant protein on non-woven fabric sheets. Colloidal gold is used as an indicator for the presence of antigens binding to the membrane on the rapid lateral flow test strip. After the proteins are bound to colloidal gold to form the conjugate, the fabric sheets are placed in a dry room for at least 2 h for drying. Subsequently, the purified antigen, diluted in PBS, is coated on the test region. Simultaneously, HCV IgG polyclonal antibody, diluted in PBS, is coated on the control region. The coated membrane is dried for a minimum of 24 h and then blocked with a particular blocking solution. Finally, the test strip is assembled such that everything slightly overlaps in order to allow for the continuous lateral flow of the liquid sample. The test strip has an absorption pad, a strip of membrane, a conjugate fibre and other fibres to hold the conjugate fibre in place. Instructions on how to assemble a test strip were provided by Artron BioResearch Inc. The combination of the coating protein, conjugate protein, conjugate fibre and dialysis buffer forms a ‘system’ in a test strip. The test strip developed was tested with different positive and negative HCV sera. I-LFIA was manufactured using IgG antibody conjugating colloidal gold instead of antigen.

### Test principle and assay procedure

**Figure 2.**
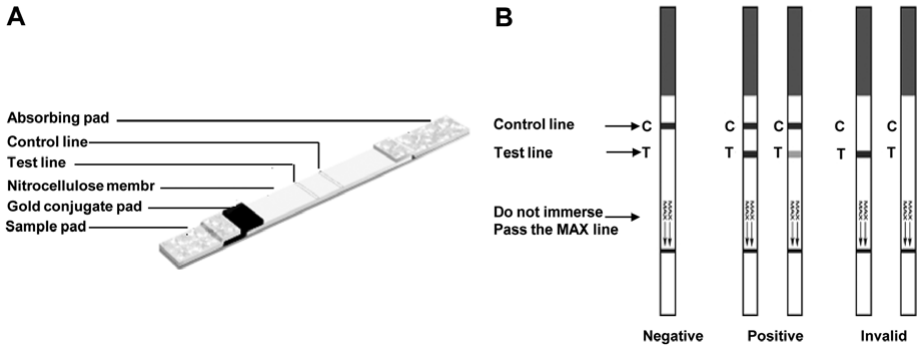
(A) Schematic diagram of a colloidal gold-based DAS-LFIA strip. The strip is composed of a sample pad, a conjugate pad that contains the core combined with NS3 (1183–1947 aa) for double antigen sandwich LFIA and antibody for indirect LFIA, and an absorbent pad attached to a nitrocellulose membrane, with a test line and an IgG control line impregnated onto the surface of the membrane. (B) Representative results of LFIA with serum. T, test line is present for samples with HCV antibody and absent for samples without HCV antibody; C, control line shows a normal flow of the liquid through the strip. Two lines indicate a positive result and one line indicates a negative result.

Each test solution (50 μl) was pipetted onto the sample pad and driven to migrate by capillary action along the strip. If HCV antibodies were present in the serum or plasma, they would react with the colloidal gold-conjugated antigen to form an antibody-antigen complex. This complex would flow through the absorbent device and bind to the antigen in the positive reaction test zone (‘T’ area), forming a gold-conjugated Ag-Ab-Ag sandwich complex, producing a pink-purple colored band. A colored band in the control region of the device indicates adequate sample volume and capillary action. The absence of a colored band in the control region is an indication of an invalid result. Positive results were read as soon as two colored bands appeared. Negative samples provided only one pink control band. If no control band was present, the test was considered invalid. Color formation for both reactions was complete after 5–10 min. A schematic representation of possible test results is shown in Fig. 2B.

### Determination of sensitivity and specificity of the HCV strip

To assess the sensitivity of the HCV strip, a panel of 23 standard positive sera were simultaneously measured by the ELISA, DAS-LFIA, I-LFIA and RT-PCR methods. In addition, serial dilutions of a reference panel with known amounts of anti-HCV antibodies with a concentration from 8 NCU/ml (NCU meaning national clinical unit) to 0.5 NCU/ml were measured by the DAS-LFIA strip. To assess the specificity and accuracy the ddH_2_0, positive enhancement sample (a set of quality control references for anti-HCV antibody detection that contains known amounts of anti-HCV antibodies; Artron BioResearch Inc.), BS control and 8 HCV-negative patients were analyzed by the DAS-LFIA strip.

### Detection of HCV antibodies in clinical specimens

A total of 300 clinical samples was analyzed by the new DAS-LFIA method as described above and the HCV ELISA test kit (Shanghai Huaguan Biochip Co., Ltd.) according to the supplier’s instructions.

### Statistical analysis

The statistical package SPSS 11.5 was used for data analysis. P-value ≤0.05 was considered to indicate a statistically significant difference.

## Results

### Construction of expression plasmids

For the expression of HCV proteins in *E. coli*, corresponding coding sequences were cloned into the histidine fusion expression vectors, pET32a(+) and pQE30, or the GST-tag expression vector, pGEX-4T-2, as shown in Table I and Fig. 1. Recombinant plasmids were examined and confirmed by PCR amplification, restriction enzyme digestion and DNA sequencing. We successfully produced a set of recombinant proteins derived from HCV structural (core, E1 and E2) and non-structural proteins (NS2, NS3, NS4A, NS4B, NS5A and NS5B) in BL21 (DE3) cells by using the *E. coli* expression system.

### Expression and purification of recombinant HCV proteins

**Figure 3.**
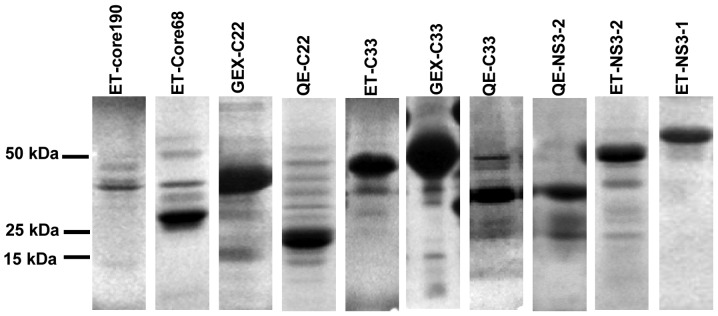
SDS-PAGE analysis of purified recombinant HCV proteins. Individual HCV genes were inserted into the His-tag or GST-tag expression plasmids. Recombinant HCV proteins were produced in BL21 (DE3) cells and purified. Samples of purified HCV proteins were separated by 10–12% SDS-PAGE and stained with Coomassie blue.

In order to obtain pure proteins, recombinant plasmids were used to transform *E. coli* BL21 (DE3) cells and expression was induced with 1 mM IPTG. The expressed proteins were purified with Ni^2+^-chelate affinity chromatography and a glutathione sepharose™ 4B column. Proteins were examined using SDS-PAGE (Fig. 3). The position of the protein bands was consistent with the expected molecular weight of the different HCV segments. Protein concentration was determined by the BCA method, using BSA as the standard. Our further analyses demonstrated that the HCV proteins were pure and that they could be used directly for the construction of the HCV DAS-LFIA strip.

### Analytical parameters of the optimized one-step strip

In the current study, we optimized the concentration of the coating antigen, the amount of colloidal gold-labelled antigen on the conjugate pad, the characteristics of the materials used, the buffer systems, the additives, and the solvents applied to ensure that the assays ran successfully over the concentration range required (Table II).

### Sensitivity and specificity of HCV DAS-LFIA strip including core and a new NS3 recombinant protein

**Figure 4.**
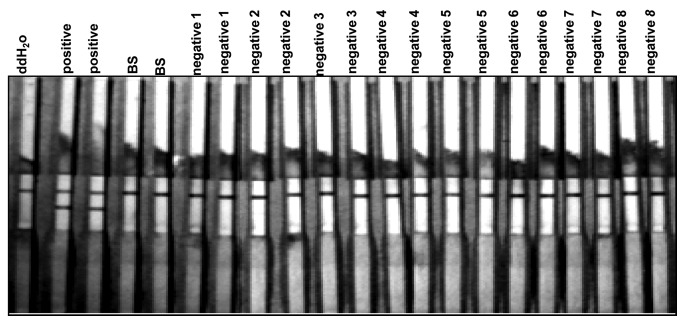
Determination of specificity of the DAS-LFIA strip.

The purified proteins would be used to determine whether they have a prognostic value in patients suffering from chronic HCV infection. Of the 23 standard positive sera (derived from confirmed HCV RNA and antibody-positive patients), 95.6% recognized the core and 95.6% recognized the NS3 protein. The remaining HCV proteins were very poorly immunogenic. Only three serum specimens recognized the NS5B protein and none of the sera recognized the NS2 protein.The results showed that the core and NS3 [1183–1476 amino acids (aa)] of the HCV polyprotein had strong positive reactions to positive sera. However, the core and NS3 (1183–1476 aa) each had one false-negative result when testing the positive control samples. Thus, we combined the core and NS3 (1183–1476 aa) as the coating antigen of the new DAS-LFIA strip. Our data suggested that the new DAS-LFIA strip was able to detect HCV antibodies at high positive rates (100%) when compared with the ELISA, I-LFIA and RT-PCR methods using the same serum samples (Table III) (P>0.05). In this model of HCV infection, the test line of the anti-HCV DAS-LFIA strip was 2 NCU/ml (Table IV). All antigens ran against negative samples did not show any signs of reactivity in comparison to the control test strip (Fig. 4). This indicated that the HCV antigen did not cross-react with other common viral antibodies and thus, was highly specific to HCV.

### Immunoassay of the 300 clinical specimens

A total of 300 samples was measured using the anti-HCV DAS-LFIA strip and anti-HCV ELISA immunoassays. In these 300 cases, we found that the rate of the anti-HCV DAS-LFIA strip and the ELISA-negative one was 78% (234/300). The rate of anti-HCV DAS-LFIA-negative but ELISA-positive was 2% (6/300). The rate of the anti-HCV DAS-LFIA-positive strip but the ELISA-negative one was 3.67% (11/300). The rate of the anti-HCV DAS-LFIA and ELISA-positive was 16.33% (49/300). The concordance between the ELISA and DAS-LFIA methods was 94.33%. The disagreement rate between the ELISA and DAS-LFIA methods was not significant (χ^2^= 0.941, P= 0.332) (Table V).

## Discussion

In this present study, we characterized the immunoreactivity of recombinant HCV polypeptides derived from many different regions of the HCV polyprotein expressed in bacteria. The purpose of synthesizing these segments is to determine which segments encoded by the genome are significant for the development of anti-HCV assays and to find a multiple epitope fusion antigen which incorporates all of the major immunodominant epitopes from the functional regions of the HCV genome. Therefore, we first constructed the different HCV segments into vectors containing His-tag or GST-tag to induce expression. The proteins were expressed in BL21 (DE3) cells as fusion proteins with a 26 kDa GST-tag or 18 kDa His-tag used for detection and affinity purification. The immunogenicity of the tagged fusion proteins was analyzed using HCV strip analysis with standard positive and negative anti-HCV sera. Our results showed that the recombinant soluble proteins were expressed successfully. The sera studied recognized the core and NS3 protein at very high levels, whereas the other proteins, such as NS4B, NS4A, NS5A and NS5B, were found to have lower levels of reactivity. The E2 protein rarely reacted with anti-HCV positive serum. Thus, the core protein in combination with a new NS3 protein (1183–1476 aa) constitutes almost all of the major immunogenic proteins of the HCV. However, this study does not exclude the possibility that the low reactivity to some HCV antigens is due to a quantitative reduction in the titer of antibodies and not due to an absence of reactivity.

To determine the contributions of various regions of the core protein and NS3 protein to infectivity diagnosis, clones of the core gene and different NS3 segments were expressed in *E. coli* cells and the recombinant proteins were used to test human anti-HCV-positive sera in the ELISA and the rapid lateral flow test strip. The data suggested that the full-length core protein and the new NS3 protein (1183–1476 aa) were suitable for analyzing the presence of antibodies against individual HCV proteins in human sera obtained from patients suffering from chronic HCV infection. These results are in agreement with those of previous studies in which the putative nucleocapsid protein (C) and non-structural proteins (NS3) were found to contain the most immunodominant epitopes (15–19).

Several studies have indicated that a peptide spanning aa 2–120 of the C gene (C22) is a major component of the commercially available second-generation anti-HCV tests. Our study demonstrates that the full-length core protein has better reactivity than C22. It is possible that the C-terminal domain of the core protein contains key peptide sequences for constructing the viral particle and regulating viral assembly (4,20).

The HCV NS3 protein is composed of an amino terminal protease and a carboxyl terminal RNA helicase (21). NS3 contains major antigenic epitopes and plays an important role in the diagnosis of HCV infection, particularly in early HCV infection (21). In this study, to our knowledge, our results demonstrate for the first time that a new NS3 segment (1183–1476 aa) shows strong reactivity in 95.6% of RT-PCR-positive samples and can act as a potential tool for the diagnosis of HCV infection.

To achieve the greatest possible sensitivity and specificity, we chose two formats of gold-based immunoassays. In the double antigen sandwich immunoassay, the HCV antigen labelled with colloidal gold is a soluble recombinant antigen. During the test, HCV antibodies in the sample react with antigen coated on the nitrocellucose membrane and gold-HCV antigen conjugates, forming a gold-conjugated Ag-Ab-Ag sandwich complex. At the same time, we carried out a series of experiments to optimize different parameters, such as the amount of immunoreagents, the type of materials, and the composition of the blocking solution and detector reagent mixture. The experimental results demonstrated that the chromatographic strip device we constructed is simple, sensitive and specific. It is an ideal test for the screening of patients with HCV infection. The presence of recombinant core and NS3 antigens in the strip may be crucial for the detection of HCV-infected patients with low antibody titers.

In conclusion, we found that recombinant antigens encoded by different HCV gene fragments display different immunoreactivity to anti-HCV antibodies. Our data show the full-length core and NS3 (1183–1476 aa) proteins have the major immuno-dominant epitopes of the HCV genome. More importantly, our study verify that the full-length core and NS3 (1183-1476 aa) recombinant antigen can be used to construct a double antigen sandwich lateral-flow immunochromatographic anti-HCV immunoassay strip. This strip allows for the more rapid and more economical detection of HCV. It also has a high sensitivity and specificity in testing for HCV. It has the potential to become a useful tool for HCV clinical detection.

## Figures and Tables

**Table I.** Sequences of oligonucleotide primers that were used to validate the expression of several different HCV segment genes.

No.	Primer sequences	Primary description	Enzyme site
F1	5′-CGGGATCCATGAGCACGAATCCTAAACC-3′	Core-190	*Bam*HI
R1	5′-TTAAGCTTCTGAAGCGGGCACAGTC-3′		*Hin*dIII
F2	5′-CGGGTACCATGAGCACGAATCCTAAAC-3′	Core-68	*Kpn*I
R2	5′-TTGGATCCACGTGCCTTGGGGATA-3′		*Bam*HI
F3	5′-CGGGTACCACGCAAGACTGCAATTGTT-3′	E1 300–407	*Kpn*I
R3	5′-TTAAGCTTGCTTGGCGCCTGGT-3′		*Hin*dIII
F4	5′-CGGGATCCTACCAAGTGCGCAATTC-3′	E1 192–318	*Bam*HI
R4	5′-CGAAGCTTATGCCATGCGATGACC-3′		*Hin*dIII
F5	5′-AAGGATCCACACCAGGCGCCAAG-3′	E2 403–642	*Bam*HI
R5	5′-TTGAATTCCGCTTCCAGCCTGTG-3′		*Eco*RI
F6	5′-CCGGTACCTTGGAGAACCTCGTAAT-3′	P7 747–807	*Kpn*I
R6	5′-TGGAATTCGTATGCCCGCTGAG-3′		*Eco*RI
F7	5′-TTGGTACCTGTGGCGGCGTTGTT-3′	NS2 817–1022	*Kpn*I
R7	5′-CGGAATTCCCACCCCTTGGAGACCAT-3′		*Eco*RI
F8	5′-TTGGTACCCAGACGAGAGGCCTCCTAG-3′	NS3 1034–1193	*Kpn*I
R8	5′-TTGGATCCGTCCACCGCCTTAGCC-3′		*Bam*HI
F9	5′-AAGGATCCGTGTGCACCCGTGGAGT-3′	NS3 1183–1476	*Bam*HI
R9	5′-GGAAGCTTGGAGCGTGGTTGTCTCAAT-3′		*Hin*dIII
F10	5′-CCGAATTCTTCAGCCTTGACCCTAC-3′	NS3 1463–1656	*Eco*RI
R10	5′-TTAAGCTTGCGTGACGACCTCC-3′		*Hin*dIII
F11	5′-TTGGTACCGTCCTGGCTGCTCTG-3′	NS4A 1665–1710	*Kpn*I
R11	5′-CGAAGCTTGGCACTCTTCCATCTCA-3′		*Hin*dIII
F12	5′-TTGGTACCCCTGGAGCCCTTGTAGT-3′	NS4B 1888–1937	*Kpn*I
R12	5′-TTGAATTCGCTCTCCGGCACGTAG-3′		*Eco*RI
F13	5′-AAGGTACCGCAGAGGAGGATGAGC-3′	NS5A 2258–2419	*Kpn*I
R13	5′-CGGAATTCGCAGCACACGACATCTT-3′		*Eco*RI
F14	5′-TTGGTACCTGGACAGGCGCACTCGT-3′	NS5B 2425–2733	*Kpn*I
R14	5′-TTGTCGACCGAGCATGGTGCAGTCC-3′		*Sal*I

F, forward primer; R, reverse primer. HCV, hepatitis C virus. The underlined sequences show the base sequences of the enzyme sites.

**Table II.** Specification of materials and parameters of the optimized DAS-LFIA strip.

Item	Specification
Membrane	High-flow NC membrane; thickness, 140 μm±20%; absorption: speed, ≥10 mm/min; size of the pore, 5–15 μm
Fiber glass	Absorbent cotton: thickness, 0.3–0.5 mm; intensity, 50±5 g/m^2^
Absorbent paper	Absorbent cotton: thickness, 0.6–0.8 mm; intensity, 270±20 g/m^2^
Colloidal gold	Particle size, 30–60 nm
Coating buffer	Tris-HCL buffer pH 8.0
Coating concentration	1 mg/ml

DAS-LFIA, double antigen sandwich-lateral flow immunoassay; NC membrane, nitrocellulose membrane.

**Table III.** Results of the positive rates of anti-HCV antibodies detected by the DAS-LFIA strip and the other methods.

Tests	Positive rate (%)
ELISA (KHB)	(22/23) 95.65
DAS-LFIA (core and NS 1183–1476 aa)	(23/23) 100
I-LFIA (core and NS 1183–1476 aa)	(22/23) 95.65
I-LFIA (core)	(22/23) 95.65
I-LFIA (NS3 1183–1476 aa)	(22/23) 95.65
I-LFIA (NS3 1192–1457 aa)	(20/23) 86.95
RT-PCR	(23/23) 100

ELISA, enzyme-linked immunosorbent assay; DAS-LFIA, double antigen sandwich-lateral flow immunoassay; I, indirect; HCV, hepatitis C virus; aa, amino acid.

**Table IV.** Lowest test limit for a positive human anti-HCV antibody detected by the DAS-LFIA strip.

Human anti-HCV antibody concentration (NCU/ml)	LFIA result
8	Positive
4	Positive
2	Positive
1	Negative
0.5	Negative

DAS-LFIA, double antigen sandwich-lateral flow immunoassay; HCV, hepatitis C virus.

**Table V.** Results from 300 plasma donor samples detected by the DAS-LFIA strip and HCV ELISA assay.

	HCV ELISA		
DAS-LFIA	Negative	Positive	Total
Negative	234	6	240
Positive	11	49	60
Total	245	55	300

χ^2^=0.941176, P>0.05. DAS-LFIA, double antigen sandwich-lateral flow immunoassay.
